# Sequence-Based Prediction for Protein Solvent Accessibility

**DOI:** 10.3390/ijms26125604

**Published:** 2025-06-11

**Authors:** Yang Yang, Mengqi Chen, Congrui Liu, Mauno Vihinen

**Affiliations:** 1 Computing Science and Artificial Intelligence College, Suzhou City University, Suzhou 215004, China; 2 School of Computer Science and Technology, Soochow University, Suzhou 215006, China; 20215227105@alu.suda.edu.cn (M.C.); 20235227115@stu.suda.edu.cn (C.L.); 3 Suzhou Key Lab of Multi-Modal Data Fusion and Intelligent Healthcare, Suzhou 215004, China; 4 Department of Experimental Medical Science, BMC B13, Lund University, SE-22 184 Lund, Sweden

**Keywords:** protein structure, amino acid accessibility, sequence-based prediction, machine learning, solubility

## Abstract

When globular proteins fold into their characteristic three-dimensional structures, some amino acids are located on the surface, while others are situated in the protein core, where they cannot interact with molecules in the environment. Predicting the degree of solubility of amino acids provides insight into the function and relevance of residues. Residue accessibility is crucial for several protein functions, including enzymatic activity, allostery, multimer formation, binding to other molecules, and immunogenicity. We developed a novel sequence-based predictor for amino acid accessibility with features derived from three-dimensional protein structures. Several machine learning algorithms were tested, and the long short-term memory (LSTM) deep learning method demonstrated the best performance; thus, it was utilized to develop the freely available SolAcc tool. It showed superior performance compared to state-of-the-art predictors in a blind test.

## 1. Introduction

Amino acids in folded globular proteins can be in contact with solvent, other molecules, or buried inside the structure. Accessibility is a fundamental property, essential, e.g., for the folding and function of proteins. Interactions depend on the availability of contacts. This applies to the binding of substrates, regulators, drugs, and other molecules, as well as allostery, immunogenicity, solubility, protein folding, and enzymatic activity. Buried amino acids cannot bind and interact with other compounds. A substantial proportion of disease-related variations affect buried protein positions [1]. Computational methods have been developed to predict the accessibility of amino acids. Most methods are based on sequence conservation or use it as one of the features for prediction (see [2]).

Since very reliable protein structure prediction methods, such as AlphaFold2, AlphaFold3, ESMFold, and RoseTTAfold [3,4,5,6], are available, one might think that properties, including residue accessibility, could be obtained from the predicted structures. This is not always feasible. Existing experimental and modeled structures cover only a small fraction of the entire protein universe for all organisms. Everybody cannot make predictions en masse. Reliable models are not available for intrinsically disordered proteins (IDPs) and regions (IDRs), and some fibrous proteins. Another area where predictions still need improvement is in terms of complexes. Although structure predictions exhibit high overall performance, the predicted structures are not always correct. The computational resources required for structural predictions form a bottleneck that prevents many large-scale studies. Predicting longer proteins is very time-consuming. Although the human proteome is among the best-studied, nonetheless, structures are not available for all human proteins and isoforms. The situation is much worse for all other organisms. Thus, there is a need for a reliable and fast tool that predicts residue accessibility without first modeling the structure.

Many methods are available to predict the accessibility of globular proteins. In addition to the position-specific scoring matrix-based, evolutionary data-utilizing predictors reviewed in [2], some tools also utilize other features. Most of these recent tools are based on machine learning (ML). The algorithms applied include neural networks in SPINE X [7], deep learning (DL) in SDBRNN [2], DeepREx-WS [8], DMVFL-RSA [9], E-pRSA [10], PaleAle 5.0 [11], Raptor-X-Property [12], SPIDER 2 [13], SPOT-1D-LM [14], SSpro/ACCpro 6 [15], and NetSurfP-2.0 [16], and gradient-boosted regression trees in PredRSA [17]. Although these methods are useful, they have some drawbacks. A systematic performance assessment has not been made. Most of the tools utilize position-specific scoring matrices (PSSMs), either alone or in combination with other characteristics. We found that the accuracy of the best tools is somewhat over 0.8, depending on the settings. There is, thus, a need for improvement.

We collected by far the most extensive set of chemical, physical, conservation, sequence neighbourhood, and other features for accessibility prediction. We also utilized a much larger dataset of unrelated proteins than any other predictor. We tested several advanced ML methods and developed the SolAcc tool, which showed superior performance in blind tests compared to previous tools.

## 2. Results and Discussion

To train a new ML predictor for the accessibility of amino acids in proteins, we obtained a systematic PISCES selection of structures [18] from the PDB based on sequence identity and structural quality criteria. To avoid problems with related proteins, we chose data with the highest pairwise sequence identity set to 25%. Sequences with identities below 25% are in the so-called twilight zone. Thus, our datasets contained structures for proteins that were likely not evolutionarily related. In total, the datasets contained 7000 proteins with 1,641,667 amino acids (Table 1). Five hundred proteins were used for validation, and another set of five hundred proteins was used for blind testing. Both these sets contained more than 100,000 amino acids. The number of proteins and amino acids is so high that we can expect them to represent the space of protein combinations well. Together with several other innovations, including an extensive set of features, thorough algorithm testing, and systematic benchmarking, we developed a new amino acid accessibility predictor.

**Figure 1:**
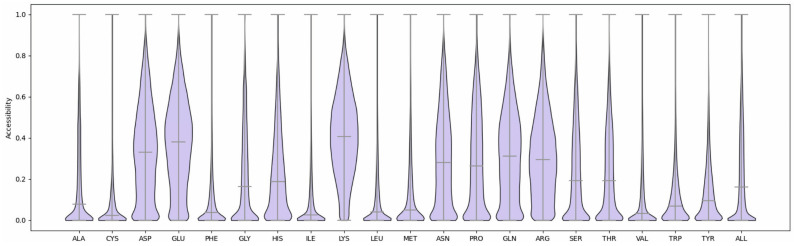
Distribution of accessibility values per amino acid type.

The distribution of amino acid accessibility in the training data is shown in Figure 1. In particular, charged and polar amino acids (D, E, H, K, N, Q, R, and S) displayed wide ranges of accessibility, while residues with non-polar side chains (A, F, L, I, M, P, V, and W) had more restricted ranges.

### 2.1. Feature Selection and Method Training

To determine whether ML tools can provide benefits in the prediction task, we defined a baseline using a linear regression model. We trained six ML algorithms to identify the best-performing one. The algorithms included random forests (RF), XGBoost, LightGBM, multilayer perceptron (MLP), residual neural network (ResNet) and long short-term memory (LSTM). At this stage, all the features were used. The results in Table 2 indicate that the algorithms exhibited somewhat different performances, although they were overall quite close to each other. The best scores were for the LSTM model. The error measures—mean absolute error (MAE), mean squared error (MSE), root mean square error (RMSE), and mean squared log error (MSLE)—were 0.1089, 0.234, 0.1530, and 0.0136, respectively. The Pearson correlation coefficient (PCC) was 0.792, and the R squared score (R^2^) was 0.5250. All six scores were the best for the LSTM-trained predictor. This algorithm was then chosen to train the final predictor, called SolAcc.

Comparison to a simpler linear regression model indicated superior performance for all the ML tools. The MAE was 0.135, the PCC was 0.615, the MSE was 0.031, the RMSE was 0.175, the MSLE was 0.018, and the R^2^ was 0.377. All these measures were better for all the ML tools.

Feature selection was performed to reduce the number of features and identify the most informative ones. We utilized the feature importance scores from LightGBM to rank 643 features and employed an incremental feature selection approach to determine the final number of features. We started with the top 10 features based on the scores and progressively increased the number of features. We compared the 5-fold cross-validated performances of the algorithms and trained several versions with different numbers of features, ranging from 10 to 643, which is the total number of features (Table 3).

The best overall performance and smallest errors were obtained when using 50 features. In the 5-fold CV, the error measures were 0.107, 0.023, 0.153, and 0.013 for the MAE, MSE, RMSE, and MSLE, respectively. The PCC was 0.740, and the R^2^ was 0.526 (Table 3).

The most important features were the relative position of the predicted site within the protein, the sequence length, and the PSSM scores for amino acids. In fact, the PSSMs for all the amino acid types were among the selected features. Thus, there were 20 PSSM features. The second largest group of features describes the neighbouring amino acids of the position of interest. There were 14 such features. There were also 14 features from the AAindex. SolAcc was then trained with 50 selected features. For definitions, see Supplementary Table S1.

**Figure 2:**
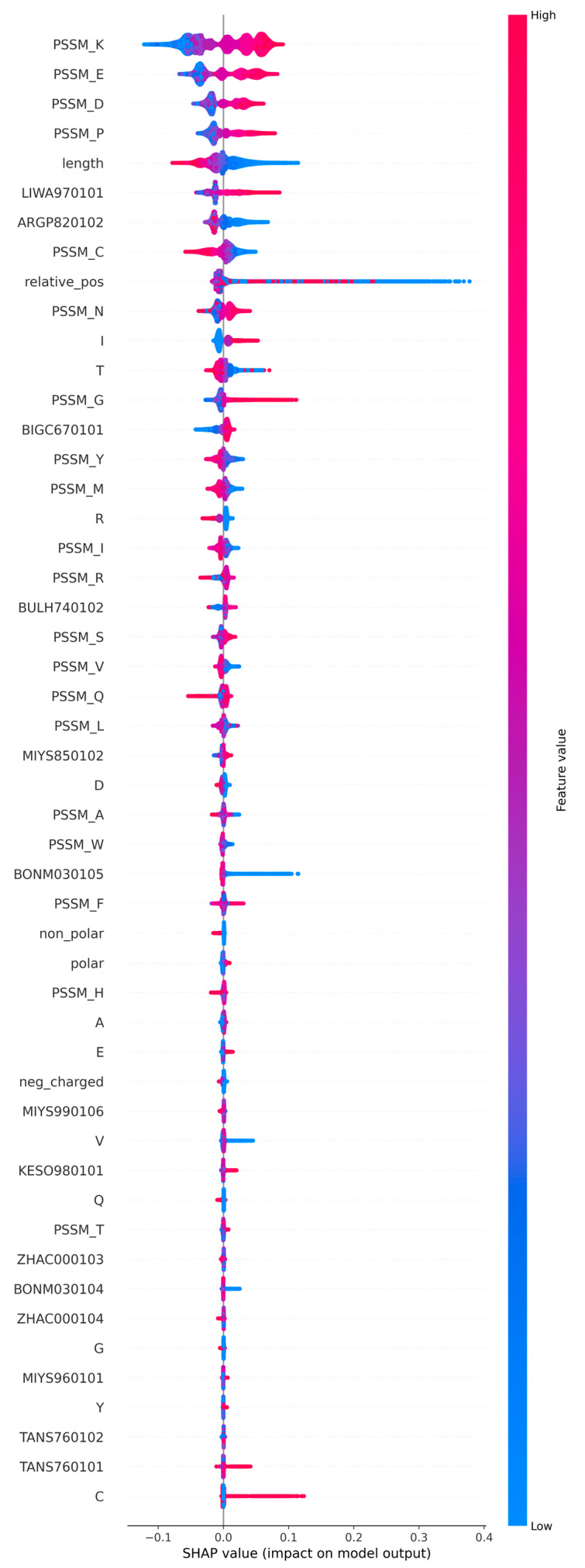
Shapley plot for the 50 selected features, organised in descending order of importance. The feature values are coloured based on their value, ranging from blue to red. The SHAP value indicates the impact of each feature on both positive and negative predictions. A positive result indicates an accessible, and negative result a buried location within the structure.

The Shapley plot (Figure 2) illustrates the importance of each feature in predicting accessibility (positive values) and buriedness (negative values). The features were arranged in order of decreasing importance. The blue colour represents a low value, and the red colour represents a high value. For binary features, those missing the feature (indicated by a 0 value) are blue, and the existence of the property is shown in red. For features with a range of values, the scale indicates the increasing feature values. PSSM scores for K, E, D, P, C, and N are among the top ten Shapley features, highlighting the importance of charged/polar and special (C) residues, which are typically at exposed positions. Sequence length, a modified version of Miyazawa–Jernigan transfer energy (LIWA970101), signal sequence helical potential (ARGP820102), and relative position within the sequence were the other top features. Several PSSM scores were among the most important features. High scores contribute to accessibility for charged or polar K, E, D, P, and others. The situation is the opposite for C, T, and Y. Shapley plots offer interpretability for ML methods. The results differ in detail from those obtained with LightGBM during feature selection. The importance of a feature in LightGBM is determined by how often it is used to split nodes. Features that are frequently used for splitting have high importance. SHAP values originate from game theory and assess the contribution of a feature to a single prediction. They can be used to compute the global importance of features. Due to the different principles, the order of the features may vary; however, the top features were consistently identified using both methods.

DL algorithms, including DeepREx-WS [8], versions of NetSurfP [16,19], SPIDER [13,20], and SSpro/ACCpro [15], have been popular among the latest accessibility predictors. The BiLSTM algorithm also performed the best in our study.

The hyperparameters in the BiLSTM were optimized and are presented in Supplementary Table S2. There were four layers with 256, 512, 1024, and 1024 units, respectively. The number of epochs was 50, and the learning rate was 0.0002. The optimizer used was Adam. The minimum sequence length was set to 32. The algorithm provides regression predictions with continuous values ranging from 0 to 1.

The performance per amino acid is presented in Supplementary Table S3. The PCC was highest for A (0.753) and lowest for W (0.514). These amino acids represent the opposite ends of the range in terms of size. Among the error measures, the lowest MAE was observed for W (0.087), while the highest was for P (0.140). The lowest MSE, RMSE, and MSLE were observed for I (0.013, 0.112, and 0.008, respectively), while the largest errors were found for P (0.033, 0.182, and 0.19, respectively). Proline is a special amino acid because it forms an internal ring with the polypeptide backbone, making it more challenging to predict. Tryptophan is the largest of the amino acids, which has many degrees of freedom in its structure.

To test the importance of sequence conservation, we compared the performance on the top 10% most conserved and 10% least similar sequences. The results are in Supplementary Table S4, showing minor but consistent improvement when more conserved sequences are predicted. For example, PCC is 0.7388 vs. 0.775 and R^2^ 0.5426 vs. 0.6014 for the bottom and top 10% of the conserved sequences, respectively. The result also indicates that SolAcc can be used over the full range of sequence similarities.

### 2.2. Comparison to Other Tools

We compared the performance of SolAcc with that of several other tools in terms of both regression and classification. E-pRSA [10], NetSurfP-3.0 [16], and SPIDER3 [20] are regression predictors, while DeepRex-Ws [8] is a classifier. To test all these tools, we had to use different setups since they provide results in different ways.

Results for the regression predictions, when comparing the predicted accessibility values to the ratio of amino acid accessibility in folded proteins, are shown in Table 4 for SolAcc, NetSurfP-3.0, PaleAle 5.0, and SPIDER3. This is the most natural performance assessment setting for SolAcc. It showed superior performance. The error scores were clearly better for SolAcc than for E-pRSA, NetSurfP-3.0, and SPIDER3. Furthermore, the R^2^ was the best for SolAcc.

The first test compared the predicted and experimental accessibility values. Some methods use different thresholds for accessibility to distinguish between accessible and buried amino acids. To investigate these tools, we compared the regression models at different accessibility thresholds. Supplementary Tables S5–S7 show the performance when using 20, 25, and 50% accessibility as the thresholds, respectively. At the 20% threshold, SolAcc was, overall, the best. At the 25% threshold, NetSurfP2.0, PaleAle5.0, and SolAcc were close, and SPIDER3 had the lowest performance. In this case, there was no clear winner, since the methods had different orders when evaluated using different measures. MCC was the greatest for PaleAle5.0, F1 for SolAcc. The situation was similar at the 50% threshold; however, the differences were larger between the tools, and they exhibited the largest discrepancies in predicting accessible and buried positions.

Based on all the comparisons in Supplementary Tables S5–S7, overall, the performance scores were most balanced when using the 20% threshold, which is the cut-off that we recommend.

### 2.3. Example Case

**Figure 3:**
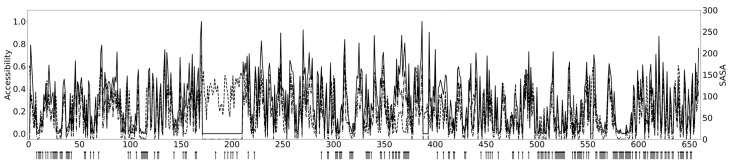
Predictions for the solvent accessibility of amino acids in BTK (constant line) and values calculated with FreeSASA (dashed line) obtained from experimental structures for the PH domain and BTK motif, SH3 domain, SH2 domain, and kinase domain. Arrows below the sequence indicate positions of XLA-causing variation.

As an example of the usage of SolAcc, Figure 3 shows the predicted and experimentally defined accessibilities of the Bruton tyrosine kinase (BTK) values calculated with FreeSASA [21] obtained from experimental structures for the PH domain and BTK motif (PDB code 1btk [22]), SH3 domain (1awx [23]), SH2 domain (2ge9 [24]), and the kinase domain (5p9j [25]). The kinase domain structure is in closed conformation, including the covalently bound inhibitor ibrutinib. The predicted and experimental structure-based accessibility is highly correlated. The figure also includes positions for known X-linked agammaglobulinemia (XLA)-causing amino acid substitutions obtained from BTKbase [26]. Many of these sites are buried. However, there are also exposed sites, such as those within the active site and the substrate binding site.

SolAcc can be used for various purposes. Residue accessibility can be correlated to other information, e.g., for binding sites and catalytic centres. In Figure 3, we can see the correlation to known disease-causing variations. Pathogenic variants are generally more likely in buried sites, especially if a larger amino acid is introduced. Such amino acids may not be accommodated in the protein structure without structural alterations, which can be detrimental, e.g., to function or stability. Combined with antigenicity predictors, the most likely immunogenic sites can be determined with improved accuracy. SolAcc will be a useful tool in many kinds of studies and predictions.

### 2.4. SolAcc Server

The SolAcc server predicts residue-specific relative solvent accessibility in proteins based solely on sequence information. Users can either manually enter or alternatively upload protein sequence(s) in FASTA format, upon which SolAcc retrieves and processes the essential sequence data. The predicted results are presented as a graph and are also available as a table. SolAcc is freely available at https://structure.bmc.lu.se/SolAcc/ and https://www.yanglab-mi.org.cn/SolAcc/. Supplementary Figures S1 and S2 show the submission page and a part of the prediction results.

## 3. Material and Methods

### 3.1. Data

We downloaded file cullpdb_pc25.0_res0.0-3.0_noBrks_len64-2048_R0.25_Xray_d2023_03_15_chains9816 from the PISCES server [18], which contains structures with a resolution better than 3.0 Å and an R-factor of <0.25 [18] (downloaded March 2023) to obtain a large, nonhomologous sequence dataset. The maximum pairwise percent sequence identity was set to 25% to exclude similar proteins. The structures of the proteins were obtained from PDB [27]. When there were several structures for a protein, we chose the longest one. A total of 7000 proteins were obtained, comprising 1,690,684 residues. The proteins were randomly divided into training, validation, and blind test sets. A total of 6000 proteins were used for training, and separate sets of 500 proteins were used for validation and testing.

### 3.2. Features

Several structure and sequence-based features were determined. In total, we had 643 features.

Residue solvent accessibilities were obtained with DSSP [28] from the structure files. The relative accessible area (rASA) was obtained by normalizing the accessible surface area (ASA) value to the maximum value of the exposed surface area of the amino acid. The sequence length and relative positions of amino acids were obtained from the sequences. A total of 596 AAindex features describe the physicochemical properties of amino acids [29]. These features are described in https://www.genome.jp/aaindex/ (accessed on 21 April 2025).

A 20-dimensional vector of neighbourhood residues was used to determine the occurrence of amino acid types within a neighbourhood in a window of 11 positions. Five additional features described the frequencies of amino acids in the window in the following five groups of amino acids: nonpolar (V, A, L, I, P, F, W, and M), polar (G, S, T, C, Y, N, and Q), charged (D, E, H, K, and R), positively charged (H, K, and R), and negatively charged (D and E).

Twenty PSSM features, one per amino acid, were derived with PSI-BLAST [30] by searching against the UniRef50 [31] database. PSI-BLAST utilized an iterative search process. The expectation value (E-value) was set to 0.001, and three iterations were performed.

### 3.3. Machine Learning Algorithms

During method development, several ML algorithms were tested, including the RF, LightGBM, XGBoost, MLP, ResNet, and LSTM algorithms. RF and LightGBM are ensemble methods. An MLP is a type of neural network, and LSTM and ResNet are commonly used in deep-learning neural networks.

RF is an extended variant of the bagging technique [32]. The algorithm divided the training dataset into several partitions and built a decision tree predictor for each partition. Finally, all the decision trees were merged into a single predictor for the final output. One of the benefits of RF is that it is not prone to overfitting.

LightGBM is a gradient-boosting framework that uses tree-based learning algorithms [33]. It is a variant of the gradient-boosted decision trees (GBDT) model with gradient-based one-side sampling (GOSS) and exclusive feature bundling (EFB), which significantly reduces the time complexity. XGBoost is an optimized distributed gradient boosting library that utilizes a parallel tree boosting approach, also known as GBDT.

A multilayer perceptron is a fully connected feedforward neural network. An MLP has several layers of input nodes connected as a directed graph between the input and output layers. Backpropagation was used in training the network. We used 5 hidden layers with 512, 256, 128, 32, and 8 nodes. The number of nodes in the output layer was 1. The activation functions of the hidden and output layers were LeakyReLU and Sigmoid, respectively.

ResNet is an artificial neural network that uses skip connections and shortcuts to avoid the problem of vanishing and exploding gradients [34]. We combined MLP or 1-dimensional convolution with ResNet, using a deeper network to train a classifier. LSTM is a form of neural network [35]. Unlike standard feedforward neural networks, LSTM also has feedback connections.

The ML algorithms utilized Python Scikit-learn scripts and employed default parameters, except for MLP, ResNet, and LSTM, which utilized the PyTorch 2.7.1 deep learning framework.

Linear regression, one of the simplest models, was used to define the baseline for predictors.

### 3.4. Feature Selection

We completed feature selection to remove redundant and uninformative features and find the most effective ones. We used an incremental feature selection method based on the importance scores. Since LightGBM required the shortest training time, it was used for feature selection. First, we trained LightGBM with a full set of features using a training dataset, from which we obtained importance scores for all 643 features and used them to rank the features. BiLSTM was trained with the top 10 scoring features and evaluated with cross-validation. Then, we added 10 features at a time and repeated the cross-validation process each time. We compared the cross-validation performances of models with different feature sets to determine the optimal subset of features.

### 3.5. Performance Assessment

We used a total of 15 measures to describe and estimate method performances.

The PCC measures the linear correlation between experimental and predicted accessibility. The PCC is the covariance of two variables divided by the product of their standard deviations; thus, it is a normalized measure of covariance, ranging between −1 and 1. The PCC reflects only a linear correlation of variables, as follows:

$$\mathrm{PCC}(Y,\;\hat{Y})=\frac{\sum_{i=1}^{N}\left(y_{i}-\mu_{Y}\right)\left({\hat{y}}_{i}-\mu_{\hat{Y}}\right)}{\sqrt{\sum_{i=1}^{N}{\left(y_{i}-\mu_{Y}\right)}^{2}}\sqrt{\sum_{i=1}^{N}{\left(y_{i}-\mu_{\hat{Y}}\right)}^{2}}},$$

where *cov* is the covariance, *σ_X_* is the standard deviation of X, *σ_Y_* is the standard deviation of Y, *μ_X_* is the mean of X, *μ_Y_* is the mean of Y, and E is the expectation.

The MAE measures the errors between paired observations as follows:

$$\mathrm{MAE}=\frac{\sum_{i=1}^{N}\left|y_{i}-x_{i}\right|}{N},$$

where *y_i_* is the prediction and *x_i_* is the actual value.

The MSE measures the average of the squares of the errors, i.e., the average squared difference between the estimated and actual values, as follows:

$$MSE=\frac{\sum_{i=1}^{N}{\left(y_{i}-{\hat{y}}_{i}\right)}^{2}}{N}.$$

The RMSE measures the difference between the values predicted by a model and the values observed. The lower the RMSE, the better the model. The RMSE is defined as follows:

$$RMSE=\sqrt{\frac{\sum_{i=1}^{N}{\left(y_{i}-{\hat{y}}_{i}\right)}^{2}}{N}}.$$

The MSLE is defined as follows:

$$MSLE\left(y,\hat{y}\right)=\frac{1}{n_{samples}}{\left(log_{e}\left(1+y_{i}\right)-log_{e}\left(1+\hat{y_{i}}\right)\right)}^{2}.$$

The R^2^ score, also known as the coefficient of determination, assesses the goodness of fit of a regression model. It indicates to what extent the model explains the variance of the target variable, representing the proportion of the target variable’s variability that the model can account for. The R^2^ score ranges from 0 to 1, where a value closer to 1 signifies a better fit of the model to the target variable. The SSR, SSE, and R^2^ score are defined as follows:

$$SSR=\sum_{i=1}^{n}{\left(\hat{y_{i}}-y^{2}\right)}^{2}$$

$$SSE=\overset{n}{\underset{i=1}{\sum }}{\left(y_{i}-\hat{y_{i}}\right)}^{2}$$

$$R^{2}=\frac{SSE}{SSR+SSE},$$

where SSE is the sum of squares error and SSR is the total sum of squares regression.

We used a confusion matrix to define seven additional measures. The cells in the matrix represent TP, true positives for positive observations predicted to be positive; FP is a false positive and represents observations predicted to be positive when they are actually negative. TN indicates a true negative, where the observation is correctly predicted as negative, and FN, false negative, is predicted as negative when it is actually positive.

The accuracy (ACC) is the proportion of the total number of correct predictions, as follows:

$$Accuracy=\frac{TP+TN}{TP+FP+TN+FN}$$

The positive predictive value (PPV) is the fraction of positive values out of the total predicted positive instances, as follows:

$$PPV=\frac{TP}{TP+FP}.$$

The negative predictive value (NPV) is the fraction of negative values out of the total predicted negative instances, as follows:

$$NPV=\frac{TN}{TN+FN}.$$

Specificity (SPE) is the fraction of negative values out of the total negative instances, as follows:

$$Specificity=\frac{TN}{TN+FP}.$$

Sensitivity (SEN) is the fraction of positive values out of the total positive instances, as follows:

$$Sensitivity=\frac{TP}{TP+FN}.$$

The F1 score is the harmonic mean of the PPV and sensitivity, as follows:

$$F1=2*\frac{PPV*SEN}{PPV+SEN}.$$

The overall performance measure (OPM) is an aggregate of six measures and is calculated as follows:

$$OPM=\frac{\left(PPV+NPV\right)*\left(SEN+SPE\right)*\left(Accuracy+\frac{1+MCC}{2}\right)}{8}.$$

The area under the curve (AUC) is under the receiver operating characteristic curve (ROC). The MCC is a reliable statistical rate that yields high scores only when the method is well-balanced and when the four cells in the confusion matrix indicate good performance, as follows:

$$MCC=\frac{TN*TN-FP*FN}{\sqrt{\left(TP+FP\right)\left(TP+FN\right)\left(TN+FP\right)\left(TN+FN\right)}}.$$

## 4. Summary

We developed a novel sequence-based predictor, SolAcc, for amino acid accessibility in protein structures. The method is based on the DL algorithm LSTM. We performed an extensive feature selection to obtain the most important features, which were used to train the final tool. SolAcc performed very well on an extensive blind test; for example, R^2^ was 0.555, MAE 0.104, MSE 0.22, and RMSE 0.148. These scores were the best among the tested algorithms. SolAcc is a versatile method that can be used for proteins from any organism and even for large datasets and proteins for which reliable structural models cannot be obtained. SolAcc can even be used to predict proteins that are not amenable to structural studies, including IDPs, IDRs, and filamentous proteins. In these cases, the users have to be cautious, since the disordered structures are extremely flexible.

## Figures and Tables

**Table 1:** Distribution of proteins to partitions.

	Sequences	Residues
Training set	6000	1,402,211
Blind test set	500	118,180
Validation set	500	121,276
Total	7000	1,641,667

**Table 2:** Performance of the methods trained with all features ^a^.

	RF	XGBoost	LGBM	MLP	ResNet	LSTM
MAE	0.123	0.126	0.120	0.120	0.128	**0.109**
PCC	0.679	0.651	0.690	0.696	0.674	**0.729**
MSE	0.027	0.029	0.026	0.026	0.028	**0.023**
RMSE	0.163	0.169	0.161	0.160	0.166	**0.153**
MSLE	0.016	0.017	0.015	0.015	0.017	**0.013**
R^2^	0.457	0.421	0.475	0.482	0.439	**0.525**

^a^ The method with the best score is indicated in bold.

**Table 3:** Feature selection performance on 5-CV of BiLSTM with different numbers of features ^a^.

	MAE	PCC	MSE	RMSE	MSLE	R^2^
10	0.115	0.705	0.025	0.158	0.015	0.496
20	0.113	0.710	0.025	0.157	0.014	0.501
30	0.113	0.715	0.024	0.156	0.014	0.508
40	0.112	0.721	0.024	0.154	0.014	0.516
50	**0.107**	**0.740**	**0.023**	0.153	**0.013**	0.526
60	0.114	0.715	0.024	0.154	0.014	0.510
70	0.113	0.712	0.025	0.157	0.014	0.508
80	0.113	0.711	0.025	0.157	0.014	0.500
90	0.112	0.720	0.024	0.155	0.014	0.515
100	0.112	0.714	0.025	0.157	0.014	0.503
200	0.110	0.728	**0.023**	0.153	0.014	0.527
300	0.110	0.729	**0.023**	0.152	0.014	0.530
400	0.109	0.734	**0.023**	**0.151**	**0.013**	**0.538**
643	0.109	0.729	**0.023**	0.153	0.014	0.525

^a^ The method(s) with the best score is indicated in bold.

**Table 4:** Comparison of SolAcc to regression predictors ^a^.

	MAE	PCC	MSE	RMSE	MSLE	R^2^
E-pRSA	0.116	**0.779**	0.024	0.155	0.015	0.513
SPIDER3	0.167	0.607	0.043	0.208	0.024	0.360
NetSufP-3.0	0.127	0.763	0.031	0.177	0.017	0.538
SolAcc	**0.104**	0.750	**0.022**	**0.148**	**0.013**	**0.555**

^a^ The method(s) with the best score is indicated in bold.

## Data Availability

The datasets used to train and test the method are freely available at the Sol-Acc websites at https://structure.bmc.lu.se/SolAcc/ (accessed on 21 April 2025) and https://www.yanglab-mi.org.cn/SolAcc/ (accessed 21 April 2025).

## Supplementary Materials

The following supporting information can be downloaded at: https://www.mdpi.com/article/10.3390/ijms26125604/s1, Supplementary Table S1. Selected features. Supplementary Table S2. Hyperparameters used for LSTM. Supplementary Table S3. Prediction performance per amino acid type. Supplementary Table 4. Performance for the top 10% and the bottom 10% or similar sequences on the test data. Supplementary Tables S5–S7. Comparisons of regression models at different thresholds. Supplementary Figure S1. SolAcc submission page. Supplementary Figure S2. The graphical display and part of the numerical predictors for the example in Figure S3.
