# Supplementary material for: Sequence-Based Prediction for Protein Solvent Accessibility
Source: Int J Mol Sci. 2025 Jun 11;26(12):5604. doi: 10.3390/ijms26125604 (PMC12193430; doi:10.3390/ijms26125604)
Supplement: Supplementary file 1 [file ijms-26-05604-s001.zip › ijms-3630600-supplementary.pdf]

SolAcc, a predictor for protein solvent accessibility

Yang Yang[1,2,3*], Mengqi Chen[2], Congrui Liu[2], Mauno Vihinen[4]

[1]Computing Science and Artificial Intelligence College, Suzhou City University, 215004, Suzhou, China, [2]School of Computer Science and Technology, Soochow University, 215006, Suzhou, China, [3]Suzhou Key Lab of Multi-modal Data Fusion and Intelligent Healthcare, 215004, Suzhou, China, [4]Department of Experimental Medical Science, BMC B13, Lund University, SE-22184 Lund, Sweden.

Supplementary Table S1. Fifty selected features used to train SolAcc.

rank	feature	description	type	Importance
1	relative_pos	Relative position in protein	Sequence	249.05
2	Length	Sequence length	Sequence	164.25
3	PSSM_P	PSSM	Evolution	159.45
4	PSSM_K	PSSM	Evolution	149.85
5	PSSM_G	PSSM	Evolution	138.20
6	PSSM_D	PSSM	Evolution	136.00
7	PSSM_E	PSSM	Evolution	122.85
8	PSSM_C	PSSM	Evolution	120.45
9	PSSM_N	PSSM	Evolution	118.65
10	PSSM_A	PSSM	Evolution	98.65
11	PSSM_R	PSSM	Evolution	95.75
12	Thr	The number of Thr	Neighbor	87.10
13	PSSM_Q	PSSM	Evolution	82.75
14	Ile	The number of Ile	Neighbor	74.30
15	PSSM_Y	PSSM	Evolution	67.80
16	PSSM_M	PSSM	Evolution	62.60
17	PSSM_S	PSSM	Evolution	61.30
18	PSSM_L	PSSM	Evolution	45.85
19	PSSM_F	PSSM	Evolution	43.50
20	Arg	The number of Arg	Neighbor	39.55
21	PSSM_H	PSSM	Evolution	39.00
22	PSSM_V	PSSM	Evolution	36.95
23	BONM030105	Distances between centers of interacting side chains in the intermediate orientation	AAindex	36.80
24	PSSM_W	PSSM	Evolution	32.75
25	Cys	The number of Cys	Neighbor	28.95
26	Val	The number of Val	Neighbor	26.70
27	LIWA970101	Modified version of the Miyazawa-Jernigan transfer energy	AAindex	26.35
28	PSSM_I	PSSM	Evolution	26.35
29	Polar	The number of polar amino acid	Neighbor	23.15

30	Ala	The number of Ala	Neighbor	18.70
31	PSSM_T	PSSM	Evolution	17.80
32	BONM030104	Distances between centers of interacting side chains in the antiparallel orientation	AAindex	17.75
33	Glu	The number of Glu	Neighbor	17.10
34	TANS760101	Statistical contact potential derived from 25 X-ray protein structures	AAindex	16.80
35	Asp	The number of Asp	Neighbor	14.15
36	Non polar	The number of non-polar amino acid	Neighbor	11.60
37	Tyr	The number of Tyr	Neighbor	11.40
38	TANS760102	Number of contacts between side chains derived from 25 X-ray protein structures	AAindex	10.60
39	Neg_charged	The number of negative charged amino acid	Neighbor	10.10
40	BIGC670101	Residue volume	AAindex	8.70
41	MIYS850102	Quasichemical energy of transfer of amino acids from water to the protein environment	AAindex	8.70
42	Gly	The number of Gly	Neighbor	8.50
43	Gln	The number of Gln	Neighbor	7.60
44	BULH740102	Apparent partial specific volume	AAindex	7.60
45	ZHAC000104	Environment-dependent residue contact energie	AAindex	7.45
46	MIYS990106	Quasichemical energy of transfer of amino acids from water to the protein environment	AAindex	7.40
47	KESO980101	Quasichemical transfer energy derived from interfacial regions of protein-protein complexes	AAindex	6.90
48	MIYS960101	Quasichemical energy of transfer of amino acids from water to the protein environment	AAindex	6.85
49	ARGP820102	Signal sequence helical potential	AAindex	6.50
50	ZHAC000103	Environment-dependent residue contact energies	AAindex	6.30

Supplementary Table S2. Hyperparameters of stacked bidirectional LSTM

Hyperparameter	Value
LSTM hidden units	256
	512
	1024
	1024
Learning rate	0.0002
Batch size	16
LSTM dropout	0.7
Epoch numbers	50
Optimizer	Adam

Supplementary Table S3. Prediction performance per amino acid type.

	MAE	PCC	MSE	RMSE	MSLE	R2
ALA	0.094	0.753	0.021	0.146	0.013	0.557
ASP	0.133	0.652	0.029	0.171	0.016	0.417
GLU	0.128	0.652	0.027	0.164	0.015	0.419
PHE	0.073	0.576	0.014	0.120	0.009	0.309
GLY	0.126	0.671	0.031	0.175	0.018	0.435
HIS	0.113	0.678	0.023	0.151	0.014	0.448
ILE	0.066	0.649	0.013	0.112	0.008	0.399
LYS	0.127	0.580	0.026	0.163	0.014	0.328
LEU	0.073	0.656	0.015	0.123	0.010	0.410
MET	0.083	0.701	0.018	0.136	0.011	0.479
ASN	0.131	0.666	0.029	0.171	0.017	0.433
PRO	0.140	0.644	0.033	0.182	0.019	0.402
GLN	0.121	0.682	0.025	0.157	0.014	0.460
ARG	0.123	0.608	0.025	0.159	0.014	0.355
SER	0.117	0.687	0.026	0.161	0.015	0.454
THR	0.113	0.690	0.024	0.155	0.014	0.466
VAL	0.074	0.689	0.014	0.120	0.009	0.455
TRP	0.087	0.514	0.017	0.130	0.011	0.212
TYR	0.094	0.545	0.018	0.134	0.012	0.239

Supplementary Table S4. Performance for the top 10% and the bottom 10% or similar sequences on the test data.

	MAE	PCC	MSE	RMSE	MSLE	R2
Top 10%	0.0964	0.7775	0.0192	0.1386	0.0111	0.6014
Bottom 10%	0.1009	0.7388	0.0209	0.1445	0.0123	0.5426

Supplementary Table S5. Comparison of regression models at the threshold of 20%.[a]

	Accuracy	F1	AUC	MCC	PPV	NPV	TPR	TNR	OPM
SPIDER3	0.715	0.672	0.718	0.454	0.815	0.659	0.572	0.864	0.382
NetSurfP-3.0	0.804	0.786	0.806	**0.624**	**0.890**	0.746	0.703	**0.909**	0.533
DeepRex-Ws	0.803	0.805	0.803	0.606	0.818	**0.788**	0.791	0.815	0.518
SolAcc	**0.813**	**0.82**9	**0.813**	**0.624**	0.845	0.782	**0.812**	0.818	**0.539**

[a]The method(s) with the best score is indicated in bold.

Supplementary Table S6. Comparison of regression models at the threshold of 25%.[a]

	Accuracy	F1	AUC	MCC	PPV	NPV	TPR	TNR	OPM
SPIDER3	0.734	0.743	0.741	0.479	0.815	0.661	0.682	0.800	0.403
NetSurfP-3.0	0.806	0.811	0.815	0.626	**0.896**	0.726	0.741	**0.889**	0.535
DeepRex-Ws	**0.823**	0.840	**0.824**	**0.644**	0.862	**0.778**	0.817	0.831	**0.556**
SolAcc	0.812	**0.847**	0.801	0.605	0.847	0.757	**0.842**	0.764	0.520

[a]The method(s) with the best score is indicated in bold.

Supplementary Table S7. Comparison of regression models at the threshold of 50%.[a]

	Accuracy	F1	AUC	MCC	PPV	NPV	TPR	TNR	OPM
SPIDER3	0.809	0.890	0.573	0.267	0.817	0.673	**0.979**	0.167	0.308
NetSurfP-3.0	0.840	0.897	0.783	**0.540**	0.914	0.602	0.881	0.685	**0.478**
PaleAle5.0	0.812	0.873	**0.805**	0.534	**0.939**	0.528	0.816	**0.793**	0.467
SolAcc	**0.879**	**0.932**	0.645	0.399	0.888	**0.695**	0.982	0.254	0.386

[a]The method(s) with the best score is indicated in bold.

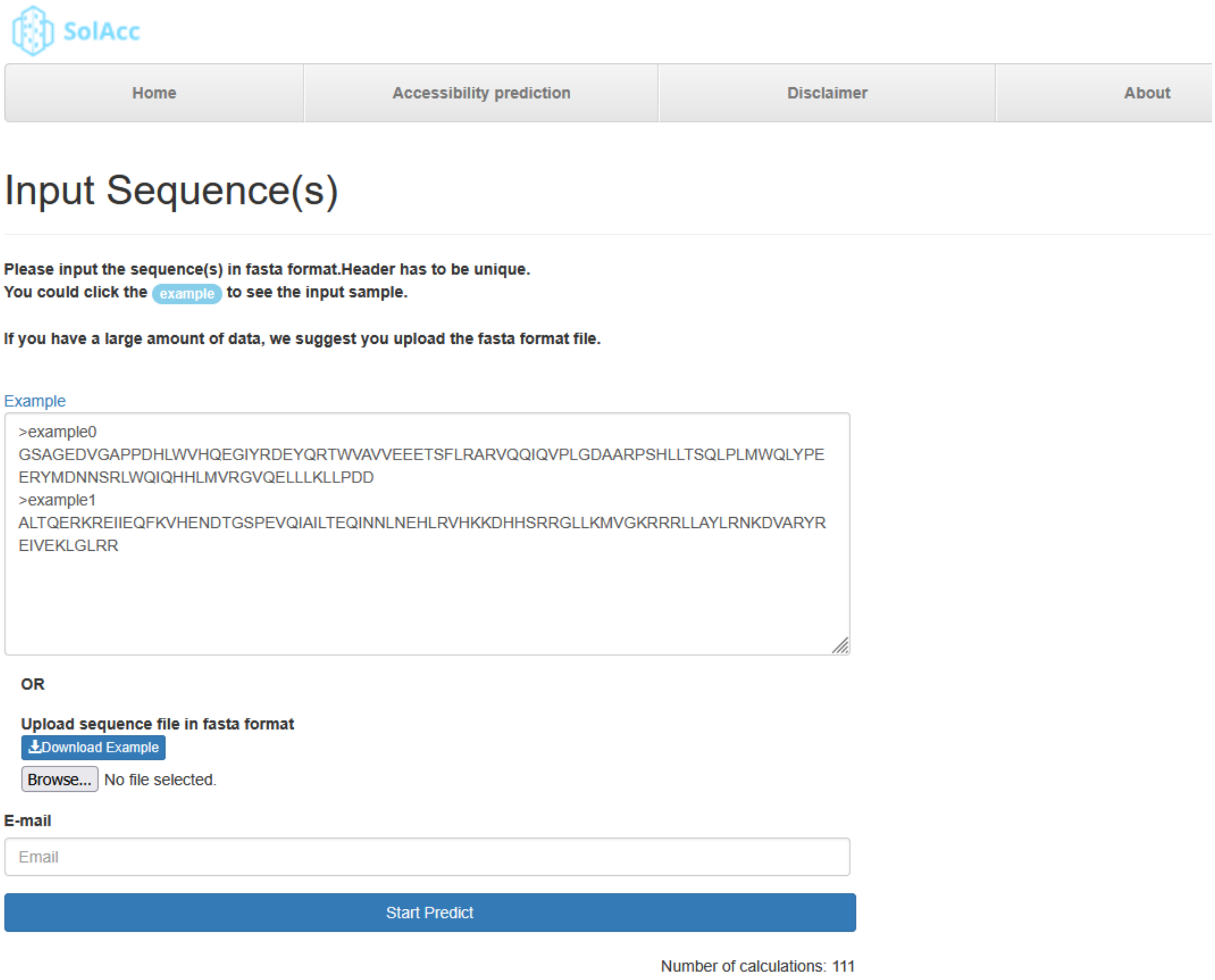

Supplementary Figure S1. Submission page of SolAcc.

Prediction of protein BRCA

record_id	aa	rsa
1	M	0.642911970615387
2	D	0.3928654193878174
3	L	0.0786086693406105
4	S	0.092887133359909
5	A	0.2869404256343841
6	L	0.2370976656675338
7	R	0.2980391979217529
8	V	0.1698439866304397
9	E	0.4412409663200378
10	E	0.2933599352836609
11	V	0.0723469853401184
12	Q	0.2359544783830642
13	N	0.4009566009044647
14	V	0.0951816216111183
15	I	0.0212793536484241
16	N	0.370694100856781
17	A	0.2664597630500793
18	M	0.0363307446241378
19	Q	0.1993296146392822
20	K	0.4336735606193542
21	I	0.1601774394512176
22	L	0.050474889576435
23	E	0.5410854816436768
24	C	0.0588662438094615
25	P	0.2422476261854171
26	I	0.0658289715647697
27	C	0.0270166154950857
28	L	0.0593819357454776
29	E	0.2516309022903442
30	L	0.0382525362074375
31	I	0.0327404141426086
32	K	0.3754336237907409
33	E	0.3316088318824768
34	P	0.2270625978708267
35	V	0.1433655172586441
36	S	0.3040453195571899
37	T	0.1183889359235763
38	K	0.4552685022354126
39	C	0.0533544905483722
40	D	0.2768138647079468
41	H	0.052372358739376

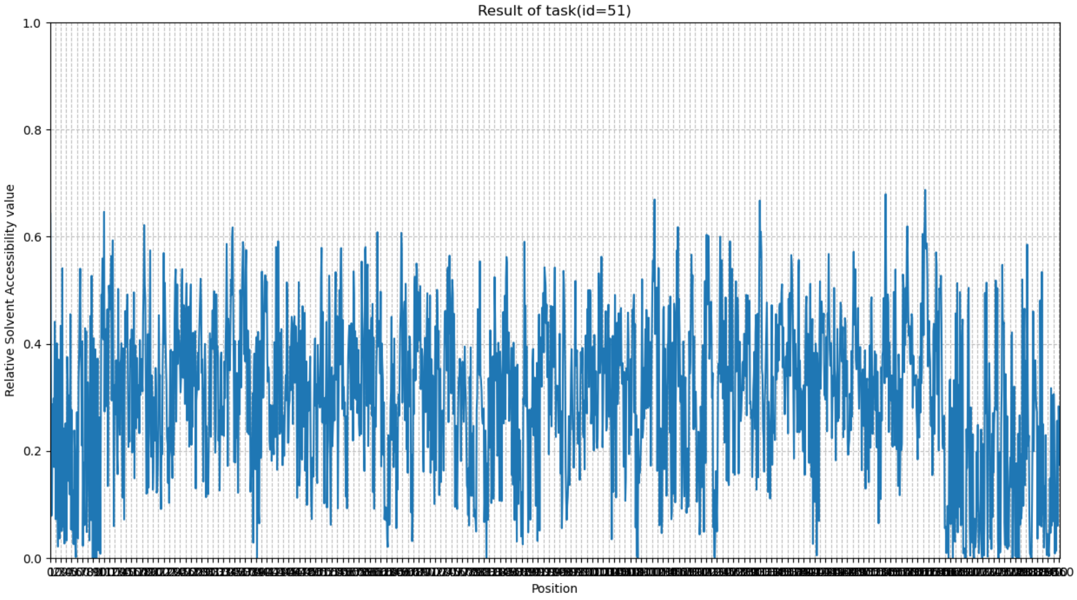

Figure S2. Example of SolAcc prediction. Part of the results for BRCA1.
